# Sorafenib-resistant hepatocellular carcinoma stratified by phosphorylated ERK activates PD-1 immune checkpoint

**DOI:** 10.18632/oncotarget.8978

**Published:** 2016-04-25

**Authors:** Jiang Chen, Tong Ji, Jie Zhao, Gaofeng Li, Jian Zhang, Renan Jin, Jinghua Liu, Xiaolong Liu, Xiao Liang, Diyu Huang, Anyong Xie, Hui Lin, Yong Cang, Xiujun Cai

**Affiliations:** ^1^ Department of General Surgery, Sir Run Run Shaw Hospital, College of Medicine, Zhejiang University, Hangzhou, Zhejiang, 310058, China; ^2^ Life Sciences Institute and Innovation Center for Cell Signalling Network, Zhejiang University, Hangzhou, Zhejiang, 310029, China; ^3^ Institute of Translational Medicine, College of Medicine, Zhejiang University, Hangzhou, Zhejiang, 310029, China

**Keywords:** hepatocellular carcinoma(HCC), phosphorylated extracellular signaling-regulated kinase(pERK), programmed death receptor-1 (PD-1), sorafenib

## Abstract

Sorafenib is a multikinase inhibitor approved as the first line treatment for late stage hepatocellular carcinoma (HCC). Due to its significant variation in clinical benefits among patients, defining prognostic biomarkers for sorafenib sensitivity in HCC would allow targeted treatment. Phosphorylated extracellular signaling-regulated kinase (pERK) was proposed to predict the response to sorafenib in HCC, but clinical supports are mixed or even contradictory. Here we found that pERK expression levels are variable in different nodules from individual patient liver. Xenografts derived from resected tumors are resistant to sorafenib inhibition when expressing low levels of pERK. This correlation of low pERK levels and sorafenib resistance is corroborated by histological characterization of chemical-induced and genetic mouse models for pERK-positive and pERK-negative HCC respectively, as well as computed tomography (CT) imaging of patient tumors with validated pERK expression. Mouse and human HCC samples expressing low pERK show strong inflammatory infiltrating cells and significant enrichment of intratumoral CD8^+^ cytotoxic T lymphocytes that express programmed death receptor-1 (PD-1). These pERK^−^PD-1^+^ patients have poorer overall and disease-free survival than pERK^+^PD-1^−^ patients. In conclusion, our data suggest that anti-PD-1 immunotherapy might complement sorafenib in treating HCC patients by targeting sorafenib-resistant cancer cells, and the dual pERK and PD-1 biomarkers would help HCC patient selection to achieve optimal clinical benefits.

## INTRODUCTION

HCC, representing 80-90% of all primary liver cancer, is the seventh most common cancer and the second leading cause of cancer death worldwide [1, 2]. Advanced HCC is treated with sorafenib, a multi-kinase inhibitor extending median survival by roughly 3 months [3]. The survival benefits vary significantly among patients due to the intrinsic genetic heterogeneity of cancer cells [4]. It is therefore imperative to identify predictive biomarkers to stratify patients most likely to benefit from the drug.

One such well studied biomarker is pERK in HCC. The level of pERK indicates the activation status of the serine/threonine kinase Raf/mitogen-activated protein kinase kinase (MEK)/ERK signaling cascade, which is directly targeted by sorafenib [5]. Inhibition of cell proliferation was shown to be dependent on the basal levels of pERK expression using patient-derived liver cancer cell lines [6]. This in vitro observation is supported by a small sample (33) of patients from a phase II study of sorafenib [7] and a slightly larger number (54) of patients in a retrospective clinical study [8], whose clinical benefits are associated with high pERK staining in their tumor samples. However, disparate results were reported that high pERK levels are associated with poor survival benefits in patients treated with sorafenib [9, 10]. Sorafenib induces tumor regression by both blocking cancer cell proliferation and tumor angiogenesis by inhibiting several tyrosine kinases including vascular endothelial growth factor receptor (VEGF)-1, 2, and 3, platelet-derived growth factor receptor (PDGF) β [5, 11]. The effectiveness of sorafenib may depend on a combined output of a myriad of signaling events. The prognostic value of pERK therefore needs further validation.

The challenge is how to translate the cell line studies [6] meaningfully to clinical significance. Tumor is a highly heterogeneous population of cancer cells that exhibit plasticity themselves and can evade targeted inhibition [12, 13]. Differentiating sorafenib-sensitive cells from sorafenib-resistant cells in HCC would allow rational design of effective combination therapy. We used chemical induced and genetic mouse models for HCC and confirmed that pERK expression levels predict sorafenib efficacy. To reduce tumor heterogeneity, we derived xenograft tumors from single tumors dissected from patients and showed unambiguously that high pERK expression correlates with sorafenib inhibition in vivo. We further found that mouse or human tumors expressing low pERK are characterized by strong inflammation and enrichment of intratumoral CD8^+^ cytotoxic T cells expressing PD-1, an immune checkpoint receptor activated to promote tumor evasion from immune clearance [14–16]. Anti-PD-1 antibodies such as pembrolizumab have recently been approved by the US Food and Drug Administration to treat late-stage melanoma [17, 18]. Our study suggests a biomarker-guided framework for designing future clinical studies combining sorafenib and anti-PD-1 immunotherapy against HCC.

## RESULTS

### High pERK level correlates with sorafenib inhibition of cell proliferation in liver cancer cell lines

**Figure 1:**
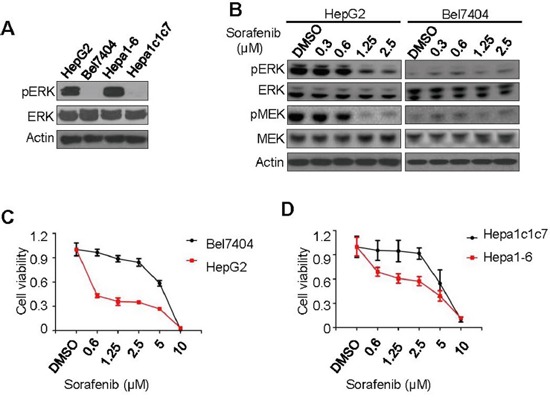
Correlation of pERK expression with sorafenib inhibition of liver cancer cell proliferation **A.** Western blot for pERK and total ERK using whole cell lysates from 4 liver cancer cell lines as indicated. **B.** Western blot for pERK and pMEK changes in HepG2 and Bel7404 cells treated with various concentrations of sorafenib. CCK-8 cell viability assays of two human **C.** and two mouse **D.** liver cancer cell lines treated with sorafenib at various concentrations for fifth day.

It was previously reported that inhibition of cell proliferation by sorafenib correlates with the basal levels of pERK in several HCC cell lines derived in-house from patient tumors [6]. To confirm this result in broadly studied cell lines, we identified two human HCC cell lines (HepG2 and Bel7404) and two mouse hepatoma cell lines (Hepa1-6 and Hepa1c1c7) as representative pERK high expression (pERK^+^) and low expression (pERK^−^) cells, respectively, by Western blot of total cell lysates (Figure 1A). When treated with sorafenib in culture, only pERK^+^ HepG2 cells, but not pERK^−^ Bel7404 cells, showed dose-dependent reduction of pERK and pMEK levels (Figure 1B). Consistently, both human (Figure 1C) and mouse (Figure 1D) pERK^+^ cells are much more sensitive to sorafenib-induced cell death than pERK^−^ cells, particularly after being treated for 4 days (Supplementary Figure 1A and IC50 in Supplementary Table 1). As reported previous [6], inactivating pERK with U0126 inhibitor desensitized HepG2 cells to sorafenib-induced proliferation arrest (Supplementary Figure 1B and Supplementary Table 1). We screened additional HCC cell lines and found that SMMC-7221, QGY-7703, and HCC-0010 cells expressed low levels of pERK (Supplementary Figure 1C) and were less sensitive than pERK^+^ cell lines to sorafenib inhibition (Supplementary Figure 1D and Supplementary Table 1). These results validate a positive correlation between pERK levels and sorafenib inhibition in liver cancer cells, although only observed in a limited number of cell lines.

### High pERK level correlates with sorafenib-induced necrosis in mouse liver cancer models

**Figure 2:**
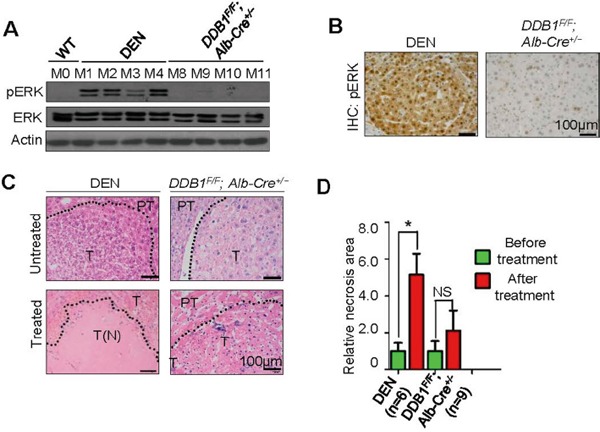
Correlation of pERK expression with sorafenib sensitivity in mouse liver tumor models **A.** Western blot for pERK in lysates of tumors dissected from DEN-induced and *DDB1^F/F^;Alb-Cre*^+/−^mouse(M) livers. **B.** Representative immunohistochemistry (IHC) staining for pERK using mouse tumor sections. Scale bar, 100 μm. **C.** H&E staining of tumor sections from mice treated or untreated with sorafenib. Dotted line indicates borders between tumor (T) and peritumor area (PT). T (N), tumor necrosis. **D.** Quantification of necrotictumor areas as in (C) (mean ± SEM; *P<0.05).

To explore the prognostic value of pERK levels in sorafenib efficacy, we took three different in vivo approaches. First, two mouse hepatoma models were identified to represent homogeneous pERK^+^ and pERK^−^ tumors. DEN, a chemical carcinogen, can induce hepatoma in mice after injected to 14-day old pups [19], and the tumors found in mice aged over 8 months were all pERK^+^, determined by Western blot of tumor lysates (Figure 2A) and immunohistochemical staining of tumor sections (Figure 2B). By contrast, tumors dissected from a genetic mouse model *DDB1^F/F^;Alb-Cre^+/−^*, in which tumorigenesis is driven by continuous hepatocyte turnover and progenitor cell activation [20, 21], express no pERK (Figure 2A and 2B). After tumors were visually confirmed and photographed after median laparotomy (Supplementary Figure 2A and 2B), both groups of mice were treated with sorafenib or PBS daily for three weeks after complete recovery from surgery. In the end, all livers were photographed and compared to surgically exposed livers before treatment (Supplementary Figure 2A and 2B). Though no tumors in either model showed gross reduction in volume, histological examination of these tumors revealed a striking increase of necrotic areas specifically in DEN-induced tumors, but not in tumors from the *DDB1^F/F^;Alb-Cre^+/−^* genetic models (Figure 2C and 2D). Intriguingly, one tumor from the genetic model almost completely disappeared after sorafenib treatment (M7, Supplementary Figure 2B). Immunostaining of the remaining tumor tissue indicated a strong pERK expression (Supplementary Figure 2C). Taken together, these animal studies suggest a strong association of pERK levels and sorafenib response in mouse liver tumor. Additional genetic mouse models for HCC could be investigated for this association.

### High pERK level correlates with sorafenib inhibition of tumor growth in patient-derived xenograft models

**Figure 3:**
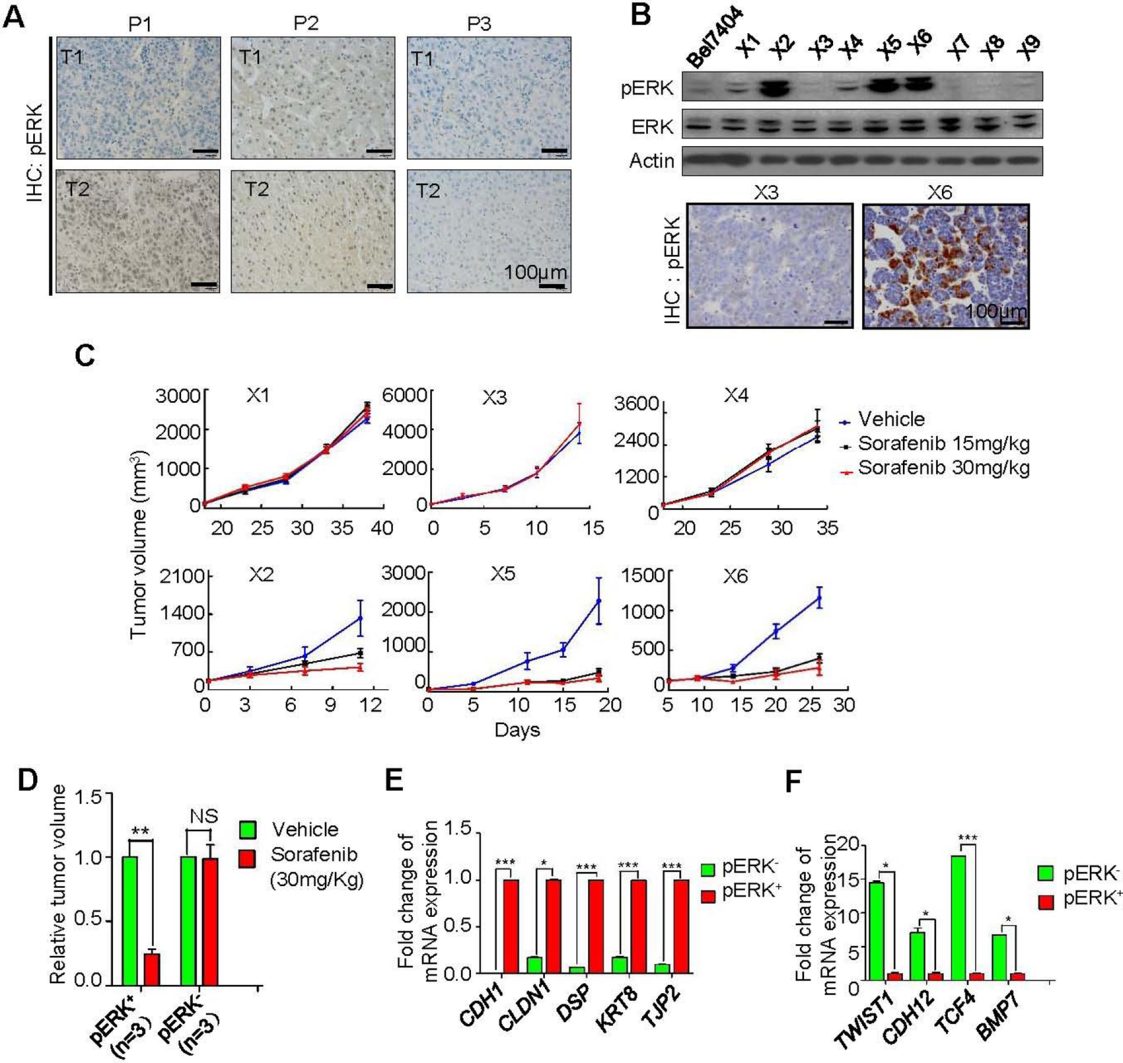
Correlation of pERK expression with sorafenib inhibition of tumor growth in patient-derived xenograft (PDX) models **A.** Representative IHC for pERK in two tumors from the same patients (P), showing distinct (P1), both high (P2) and both low (P3) expression patterns, **B.** Representative Western blot (top) and IHC (bottom) for pERK in some established PDX tumors (X). **C.** Tumor volume changes in 6 selected PDX model with different pERK expression received 15 or 30 mg/kg sorafenib treatment after the xenograft volumes reached 50-100 mm^3^. **D.** Quantification of relative tumor volumes in the 30 mg/kg sorafenib group at the end of treatment. Differential expression of epithelial markers **E.** and mesenchymal markers **F.** between pERK^+^ and pERK^−^ PDX tumors by RNA sequencing.

Unlike hepatomas from these mouse models, human liver tumors express different levels of pERK even between different nodules of the same patient (Figure 3A). Therefore, sorafenib benefits in pERK^−^ guided treatment would be complicated despite a possible prognostic value of pERK expression, and this might explain the discrepancy in patient survival studies [7–10]. To examine the efficacy of sorafenib on individual tumors, we generated patient-derived xenografts using surgically removed HCC samples and passaged them in nude mice for up to three cycles. These xenograft tumors expressed either high or low pERK (Figure 3B). Treating mice bearing these xenografts with 15 or 30 mg/Kg sorafenib resulted in a dramatic growth arrest of only pERK^+^ xenografts in volumes (Figure 3C and 3D). The weight ratio of treated over control xenograft tumors, dissected at the end of the treatment, was significantly lower in pERK^−^ PDX group than pERK^+^ group (0.24±0.04 vs.0.98±0.12, respectively; P=0.0004) (Supplementary Table 2 and Supplementary Figure 2D), which is consistent with the results observed in cell lines (Figure 1) and mouse models (Figure 2), and reinforces the predictive value of pERK levels in sorafenib response. RNA sequencing of these patient xenograft tumors (Supplementary Table 3) revealed an enrichment of epithelial markers such as *CDH1*, *CLDN1*, *DSP*, *KRT8* and *TJP2* in pERK^+^ xenografts (Figure 3E), and mesenchymal markers such as *TWIST1*, *CDH12*, *TCF4* and *BMP7* in pERK^−^ xenografts (Figure 3F), suggesting that epithelial-mesenchymal transition (EMT) might account for the loss of sorafenib sensitivity in pERK^−^ HCC xenografts.

### High pERK level correlates with sorafenib-induced shrinking of individual HCC nodules

**Figure 4:**
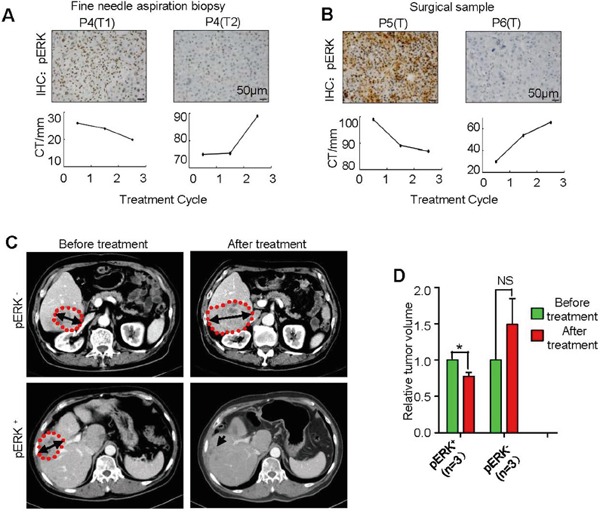
Correlation of pERK expression with individual tumor size change in HCC patients treated with sorafenib **A.** Representative pERK IHC of needle aspiration biopsies from two tumor nodules of the same patient (top panel), and computed tomography (CT) measurement of tumor maximum diameter changes after each of the three sorafenib therapy cycles (bottom panel). Scale bar, 50 μm. **B.** IHC of surgically removed samples from two patients (top) and their tumor size follow-up with CT (bottom). **C.** Representative CT scan pictures of tumor size changes before and after sorafenib therapy. Dotting red lines outline the tumor nodules. **D.** Quantification of tumor size changes as in (C) (mean + SEM; *P<0.05; n= 3).

To follow the direct effect of sorafenib on patient tumor volumes, we identified three pairs of patients with HCC nodules that were confirmed to express either high or low pERK by immunostaining the needle aspiration biopsy or surgical samples (Figure 4A and 4B). These patients received 400 mg of sorafenib twice daily, and their tumor individualnodule sizes were imaged and measured by CT scanning every 2 months during continuous sorafenib treatment (Figure 4C). In these limited number of samples, all pERK^+^ nodules exhibited various degree of size reduction after treatment, while pERK^−^ nodules were not responsive (Figure 4A, 4B and 4D). It is particularly striking that two tumor nodules found in the same patient liver responded differently to sorafenib inhibition according to their pERK expression levels (Figure 4A).

Based on the positive correlation of pERK levels and sorafenib effectiveness in cancer cell lines, mouse models, patient-derived xenograft models, and by patient tumor imaging, we conclude that pERK is a strong prognostic biomarker candidate to predict sorafenib treatment effectiveness. Given the heterogeneity of pERK expression in different tumors from even the same patient liver, successful HCC management would require a better understanding of pERK^−^ tumors for rational design of combination therapy with sofarenib.

### Liver tumor with low pERK level shows increased inflammatory PD-1^+^CD8^+^ T cell infiltration

pERK expression levels in 104 HCC samples were evaluated by immunostaining, and only 35 (33.7%) of them were found to express high levels of pERK (data not shown). This implies that sorafenib would not benefit most HCC patients if pERK can be validated as such a predictive marker in future large scale of clinical studies. It is therefore imperative to identify unique molecular signature in pERK^−^ HCC that can be therapeutically targeted. We reported previously that hepatomas, which are pERK^−^ (Figure 2A), from the *DDB1^F/F^;Alb-Cre^+/−^* mouse model were characterized by strong inflammatory infiltration [20]. The inflammation was much weaker in pERK^+^ DEN-induced mouse tumors, as demonstrated by the presence of less number of F4/80^+^ macrophages and CD45^+^ lymphocytes (Supplementary Figure 3A and 3B). To understand the sub-populations of these inflammatory cells, co-immunofluorescent staining of the tumor sections revealed a significant enrichment of PD-1^+^CD8^+^ T lymphocytes in pERK^−^ hepatomas as compared to those in pERK^+^ hepatomas (Supplementary Figure 3C and 3D).

**Figure 5:**
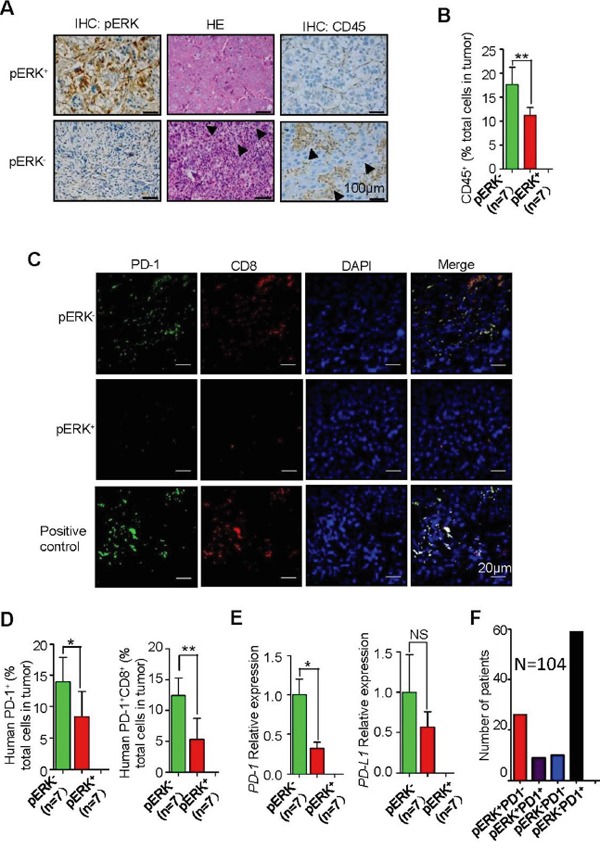
Increased inflammation and intratumoral PD-1^+^CD8^+^ T lymphocytes in pERK^−^ HCC samples **A.** Representative IHC for CD45 in pERK^+^ and pERK^−^ human HCC samples. Arrowheads indicate inflammatory clusters. **B.** Quantification of CD45^+^ cells in human HCC samples. n=7. **C.** Representative co-IF staining for PD-1 and CD8 in human HCC samples. **D.** Quantification of the percentage of PD-1^+^ cells (*P<0.05; n=7). and PD-1^+^CD8^+^ cells (**P<0.01; n=7) in sections. **E.** Real-time PCR analysis of PD-1 (**P<0.01; n=7) and PD-L1 (P=0.817; n=7) mRNA levels in human HCC tissues. **F.** Distribution of pERK and PD-1 markers in a total of 104 patient samples.

The lymphocyte infiltration (Figure 5A and 5B) and PD-1^+^CD8^+^ T cells (Figure 5C and 5D) were also found to be more abundant in most pERK^−^ human HCC samples than in pERK^+^ samples. Quantitative real-time PCR analysis of total tumor mRNA confirmed an increase of *PD-1* transcript levels but not *PD-L1* levels (Figure 5E) or inflammatory cytokines such as *tumor necrosis factor α* (*TNF α*) (Supplementary Figure 4A) and *interleukin-6* (*IL-6*) (Supplementary Figure 4B). In addition, profiling of PD-L1 expression in liver cancer cell lines (Supplementary Figure 4C) and human HCC samples (Supplementary Figure 4D and 4E) did not show a correlation between pERK and PD-L1 levels. Although pERK expression is in general inversely correlated with the abundance of PD-1^+^ T cells, pERK^+^PD-1^+^ and pERK^−^PD-1^−^ tumors were also identified (Supplementary Figure 3E). In a larger screening of 104 HCC samples, 57% tumors are pERK^−^PD-1^+^, 25% pERK^+^PD-1^−^, 10% pERK^−^PD-1^−^, and 8% pERK^+^PD-1^+^ (Figure 5F).

To explore the potential mechanism, two mouse HCC cell lines, pERK^+^ Hepa1-6 and pERK^−^ Hepa1c1c7, were implanted to immunocompetent C57BL/6 mice subcutaneously. The tumors were harvested and the *PD-1* expression were analyzed by RT-qPCR and immunofluorescent staining. As expected, *PD-1* mRNA and PD-1^+^ cells were more abundant in pERK^−^ tumor than pERK^+^ tumor (Supplementary Figure 5A and 5B), suggesting that pERK^−^ cancer cells might more effectively recruit PD-1^+^ cells than pERK^+^ cancer cells. Whether pERK expression is the only factor determining the PD-1^+^ cell abundance needs further studies.

### pERK and PD-1 expression in HCC tissues associates with HCC progression

**Figure 6:**
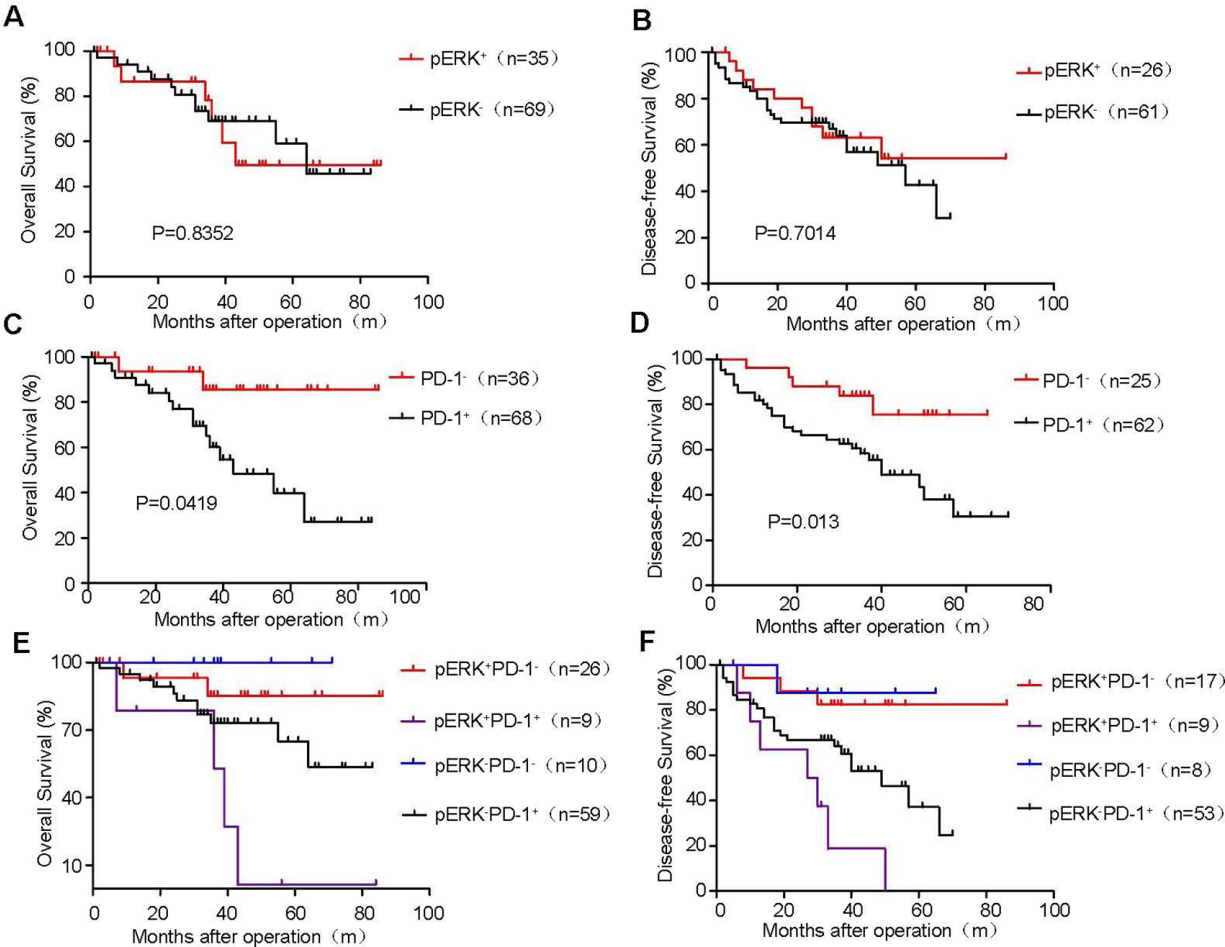
Kaplan–Meier survival curves of postoperative HCC patients stratified by pERK and PD-1 expression Overall survival of 104 HCC patients grouped by pERK expression levels **A.** PD-1^+^ cell abundance **C.** and different combination of pERK and PD-1 levels **E.** Disease-free survival of 87 patients by pERK expression levels **B.** PD-1^+^ cell abundance **D.** and different combination of pERK and PD-1 levels **F.** P values are indicated.

To better stratify HCC patients for potential sorafenib treatment or anit-PD-1 immunosuppression, we evaluated the association of dual pERK and PD-1 expression patterns in HCC samples with the prognosis of patients. We analyze 104 patients in OS grounp, among whom only 87 patients were monitored by bimonthly CT scanning to determine DFS. No obvious overall survival or disease-free survival benefits were found in either group based on pERK expression levels (Figure 6A and 6B). However, high PD-1 expression in HCC tissues (2/3 of total patients) was significantly correlated with poor overall survival (Figure 6C) and poor disease-free survival (Figure 6D), regardless of the status of pERK expression. Among these PD-1^+^ HCC patients, pERK^+^ HCC patients (9 out of a total of 68) suffered the worse overall survival (Figure 6E) and disease-free survival (Figure 6F). This small population of pERK^+^PD-1^+^ patients are however more likely to respond favorably to combination treatment with sorafenib and anti-PD-1 monoclonal antibody or to anti-PD-1 as an adjuvant therapy. By contrast, more than 85% PD-1^+^ HCC patients expressed little pERK and would be expected to benefit less if any from sorafenib therapy. Our dual stratification analysis should allow rational design of clinical trials to achieve targeted therapy without inducing unnecessary sorafenib-induced complication.

## DISCUSSION

Sorafenib targets multiple kinases and inactivates the downstream ERK signaling in liver cancer cells. High levels of pERK would indicate the activation of this survival pathway in liver tumors, justifying the use of pERK expression as an intuitive biomarker to predict the sorafenib efficacy. Here we present evidence in cancer cell lines, mouse models, and patient tumors about a significant correlation between high pERK expression and effective sorafenib inhibition (Figures 1–4). However, several clinical studies failed to correlate the pERK expression with HCC progression in patients treated with sorafenib [7–10]. We found that pERK expression can be variable between distinct tumor nodules of the same patient (Figures 3A and 4A). Sorafenib was approved as a first line treatment for advanced HCC patients who typically develop multiple tumor nodules. It is therefore possible that the survival benefits of sorafenib-treated advanced HCC patients are complicated by the tumor heterogeneity in pERK expression.

PD-1 is an immune checkpoint inhibitor primarily expressed in CD8^+^ T lymphocytes, and often co-opted by cancer cells to escape immune surveillance. Anti-PD-1 antibody can block this checkpoint and induce regression of several tumor types including HCC in preclinical studies [22, 23]. Using orthotopic xenograft and genetic mouse models, Chen et al. reported that an antibody blocking PD-1 effectively inhibits tumor growth but shows no additional inhibition when combined with sorafenib [24]. It will be interesting to determine if tumors from the two models are pERK^−^, consist with their lack of response to sorafenib treatment [24]. Based on our results, therapeutic benefits of combinatorial treatment with anti-PD-1 antibody and sorafenib could be best exemplified in pERK^+^PD-1^+^ HCC patients, a minor population of all affected (less than 10%) but with the worst postoperative recurrence (Figure 6). Most patient tumors are pERK^−^PD-1^+^ (60 out of a total of 104), and thus not expected to respond well to sorafenib and can be subjected to anti-PD-1 immunotherapy alone. PD-1^−^ HCC patients have significant better overall and disease-free survival than PD-1^+^ HCC patients, as reported previously [25], and additional pERK stratification does not find significant improvement in survival in subgroups. Since many cancer patients fail to respond to immunotherapies, biomarkers such as pERK and PD-1 in HCC should be able to help identify the most susceptible cancer patients for clinical trials and personalized treatment [26].

## MATERIALS AND METHODS

### Patients, HCC tissues and their characterization

Two series of HCC patients were used in the current study. One series included 9 randomly selected patients receiving sorafenib treatment. 5 of these patients underwent laparoscopic liver resection and 4 fine needle aspiration biopsy between February and December 2010 at the Sir Run Run Shaw Hospital (Zhejiang University, Hangzhou, China). There were no other former or postoperative treatment of the 3 pairs of patients selected for CT scan received only sorafenib therapy with no prior or additional postoperative treatment. The other series included 186 randomly selected HCC patients who underwent liver resection between January 2008 and December 2012. Those patients who received chemotherapy or radiotherapy before sampling were excluded. Among the 186 cases, 64 were excluded due to the loss or poor maintenance of the tissue blocks, and 18 excluded due to lack of follow-up records. In the end, 104 cases were determined for the pERK and PD-1 IHC staining on tissue sections. The minimum follow-up time was 12 months and the median follow-up time was 43 months (range, 12–75 months). The overall survival and recurrence was determined by survival analysis. The use of human samples was approved by the medical ethical committee of Sir Run Run Shaw Hospital. Written informed consent was obtained from each patient.

The detailed demographic and clinical characteristics of these patients were shown Supplementary Table 4. The median age was 50 years (17 to 80 years range). Hepatitis B virus infection was the predominant etiology. One hundred and eighty (180) patients (95%) were characterized as Child-Pugh A class. At the baseline, one patient had portal vein invasion, and 6 patients had micrometastasis. Two patients had Barcelona Clinic Liver Cancer (BCLC) stage B tumors, and one patient had stage C tumors.

### Chemicals and other reagents

For in vitro experiments, sorafenib (Bayee Biotech, Shanghai) was dissolved in DMSO, and the final concentration of DMSO in cell culture was kept below 0.1%. For animal experiments, sorafenib was administered daily by oral gavage at 15 mg/kg or 30 mg/kg, in which sorafenib was dissolved in a 50% cremophor EL (Sigma, St Louis, Mo) −50% ethanol mixture and sonicated for 5-10 minutes. Once in the solution, the aqueous fraction (75% water) was diluted to produce the final dosing solution [27]. Sorafenib was stored in dry form away from light and prepared immediately prior to use.

### Cell culture

HepG2, hepa1c1c7, hepa1-6, hep3B, SMMC-7721, QGY-7703, K562 and HL-7702 were purchased from ATCC in 2010. 0005 and 0010 were patient derived cell lines from Wu Xi App Tec Co. Ltd. in 2012. Bel7404 was a gift from Wu Xi App Tec Co. Ltd. HepG2, hepa1c1c7, hepa1-6, hep3B, K562, and HL-7702 were authenticated by STR analysis in the past 0.5-1 year. The other three cell lines have not been tested.

All cells were grown in vendor-specified culture medium supplemented with 10% fetal bovine serum, and maintained in an atmosphere of 5% CO2 in a humidified 37°C incubator. Cell proliferation was analyzed according to the CCK-8protocol (Dojindo, Kumamoto, Japan).

### Animal models

DEN-induced model and genetic mutant, *DDB1^F/F^; Alb-Cre^+/−^*, for liver tumor in mice were generated as described previously [19, 20]. Sorafenib (30 mpk) or PBS was administered by oral gavage daily for 21 consecutive days. Patient-derived xenograft (PDX) models were established from freshly dissected patient tumor fragments by implanting and passaging subcutaneously in nude mice (female, age 6–8 weeks). Treatment on PDX models starts when xenograft tumors are 50-100 mm^3^. Tumor volume was calculated using the following formula: volume = longest tumor diameter x (shortest tumor diameter)^2^/2. All mice were maintained according to the Guide for the Care and Use of Laboratory Animals published by the NIH and following animal protocols approved by the university committee. Mice were provided with sterilized food and water ad libitum and housed in negative pressure isolators with 12-hour light/dark cycles.

### Western blot

For whole protein extracts, tumor tissues or cell pellets were homogenized in RIPA buffer (Sigma) containing protease inhibitor cocktail (Roche), incubated on ice for 30 minutes, and then centrifuged for 15 minutes at 4°C at 12,000g. Primary antibodies used for Western blot include anti-Phospho-p44/42 MAPK (Erk1/2) (Thr202/Tyr204) (Cell Signaling Technology, 1:2000), anti-p44/42 MAPK (Erk1/2) (Cell Signaling Technology, 1:2000), anti-Phospho-MEK (Cell Signaling Technology, 1:2000), anti-MEK (Cell Signaling Technology, 1:2000), anti-actin (Cell Signaling Technology, 1:2000), anti-PD-L1 (Abcam, Cambridge, MA, 1:100).

### Immunohistochemistry (IHC) and immunofluorescence (IF)

IHC and IF were performed as described previously [28]. Briefly, tissue samples were processed for paraffin embedding and 5μm sections were prepared. After blocking endogenous peroxidase activity and non-specific staining, the sections were incubated overnight at4°C with primary antibodies. Antibodies used for staining include anti-Phospho-p44/42 MAPK (Erk1/2) (Thr202/Tyr204) (Cell Signaling Technology, 1:200) and anti-F4/80, anti-CD45, anti-PD-1 and anti-PD-L1 (all from Abcam, 1:100). Three different view fields were randomly selected on each slide, 100 cells were counted in each view field, and the positive cells were calculated as percentage of the total. Each slide was scored based on staining intensity (0, 1, 2, and 3 for negative, mild, moderate, and strong, respectively) and the number of stained cells (0 for negative, 1, 2, 3, and 4 for the number of stained cells ≤10%, 10-33%, 33-66%, and >66%, respectively). Scores for staining intensity and the number of stained cells were combined, with a score≤ 2 considered to be negative expression and a score ≥3 considered to be positive expression. All specimens were analyzed by a trained pathologist (Z. J.) blinded to any clinical information.

### Quantitative real-time PCR

Total RNA preparation, reverse transcription, and real-time PCR were performed as described previously [28] with the following changes. PCR was performed on a MyiQ 2 Two-Color Real-Time PCR Detection System (Bio-Rad), using the following amplification conditions: 5 min at 95°C, followed by 40 cycles of 10 sec at 95°C, 31 sec at 60°C, and 31 sec at 72°C. All assays were carried out in triplicates. Cycle threshold (CT) values were determined using the iQ5 software (Bio-Rad). Gene expression in each sample was normalized to the house keeping gene (GAPDH) expression. Relative quantification of target gene expression was evaluated using the comparative CT method. Sequences of all primers are listed (Supplementary Table 5).

### Statistic analysis

The GraphPad Prism 5 software was used to calculate statistical significance. Data were represented as the mean ± SEM. Comparisons between two groups were performed using an unpaired student's *t*-test. The survival rates were calculated using the Kaplan–Meier method, and differences were evaluated using the log-rank test. P<0.05 was considered statistically significant.

## SUPPLEMENTARY FIGURES AND TABLES


