# Supplementary material for: Sorafenib-resistant hepatocellular carcinoma stratified by phosphorylated ERK activates PD-1 immune checkpoint
Source: Oncotarget. 2016 Apr 25;7(27):41274–84. doi: 10.18632/oncotarget.8978 (PMC5173058; doi:10.18632/oncotarget.8978)
Supplement: Supplementary file 1 [file oncotarget-07-41274-s001.pdf]

SUPPLEMENTARY FIGURES AND TABLES

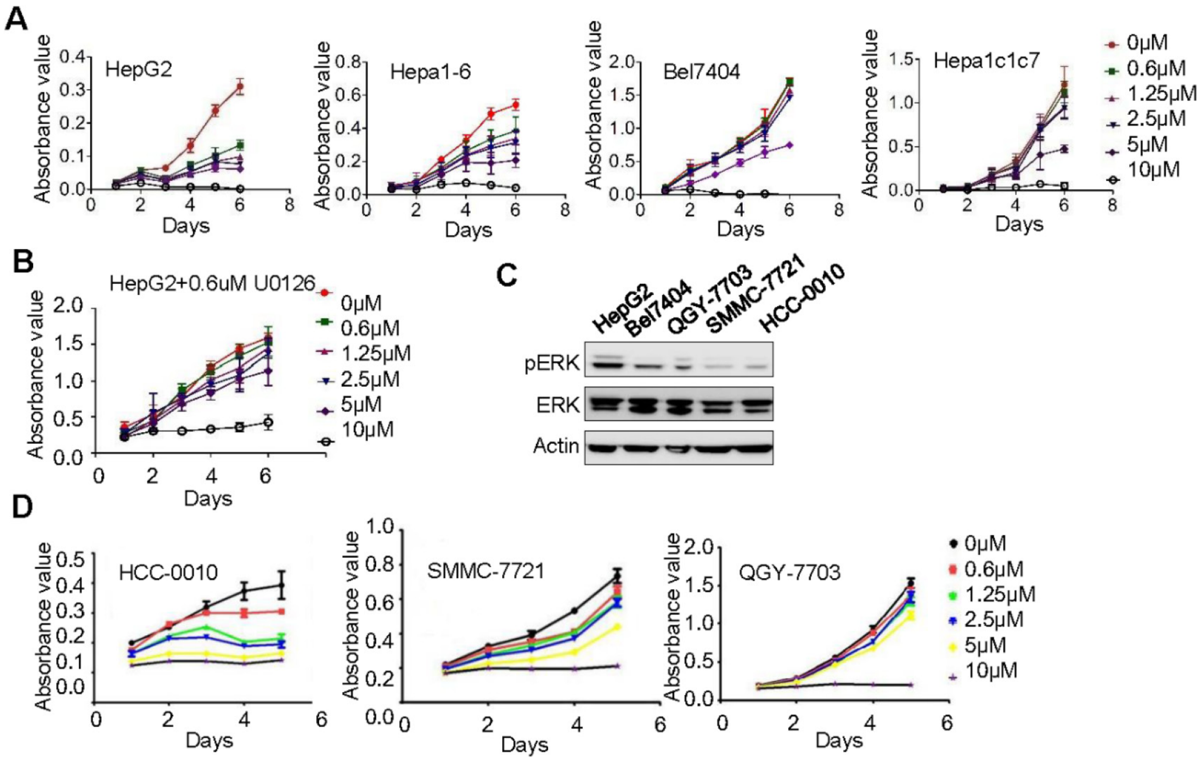

Supplementary Figure S1: Survival of liver cancer cell lines treated with sorafenib. CCK-8 cell viability assays of five human and two mouse **A** and **D.** liver cancer cell lines treated with sorafenib at indicated concentrations for up to 6 days. **B.** CCK-8 cell viability assays of HepG2 cells treated with sorafenib at indicated concentrations for 6 days, after incubation with 0.6μM U0126 for 6 hour. **C.** Western blot for pERK and total ERK using whole cell lysates from 5 liver cancer cell lines as indicated.

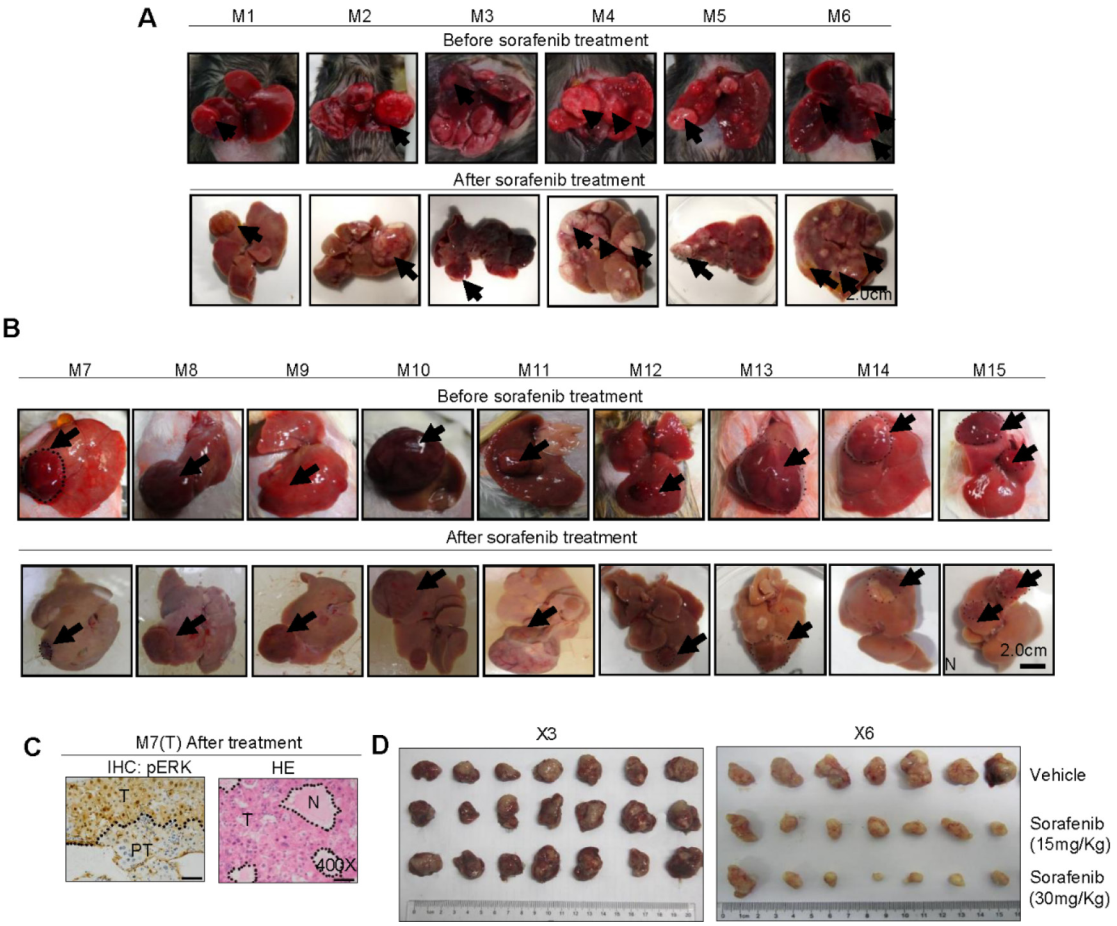

Supplementary Figure S2: Sorafenib response in mouse hepatoma models. A. Photographs of 6 DEN-induced mouse liver tumors exposed by median laparotomy before (upper) and after (bottom) 3 weeks of daily sorafenib treatment. Arrows indicate the same tumor nodules prior to and after the treatment. **B.** Photographs of 9 aged *DDB1*$^{F/F}$;*Alb-Cre*$^{+/-}$ livers before (upper) and after (bottom) sorafenib treatment, as performed in (A). **C.** Representative IHC for pERK in M7 tumor region showing strong pERK staining in the remnant tumor tissue. N, necrosis; T, tumor; PT, peritumor. **D.** Photographs of xenograft tumors dissected from pERK$^-$ X3 and pERK$^+$ X6 PDX models at the end of the treatment with vehicle, 15 mg/kg or 30 mg/kg sorafenib.

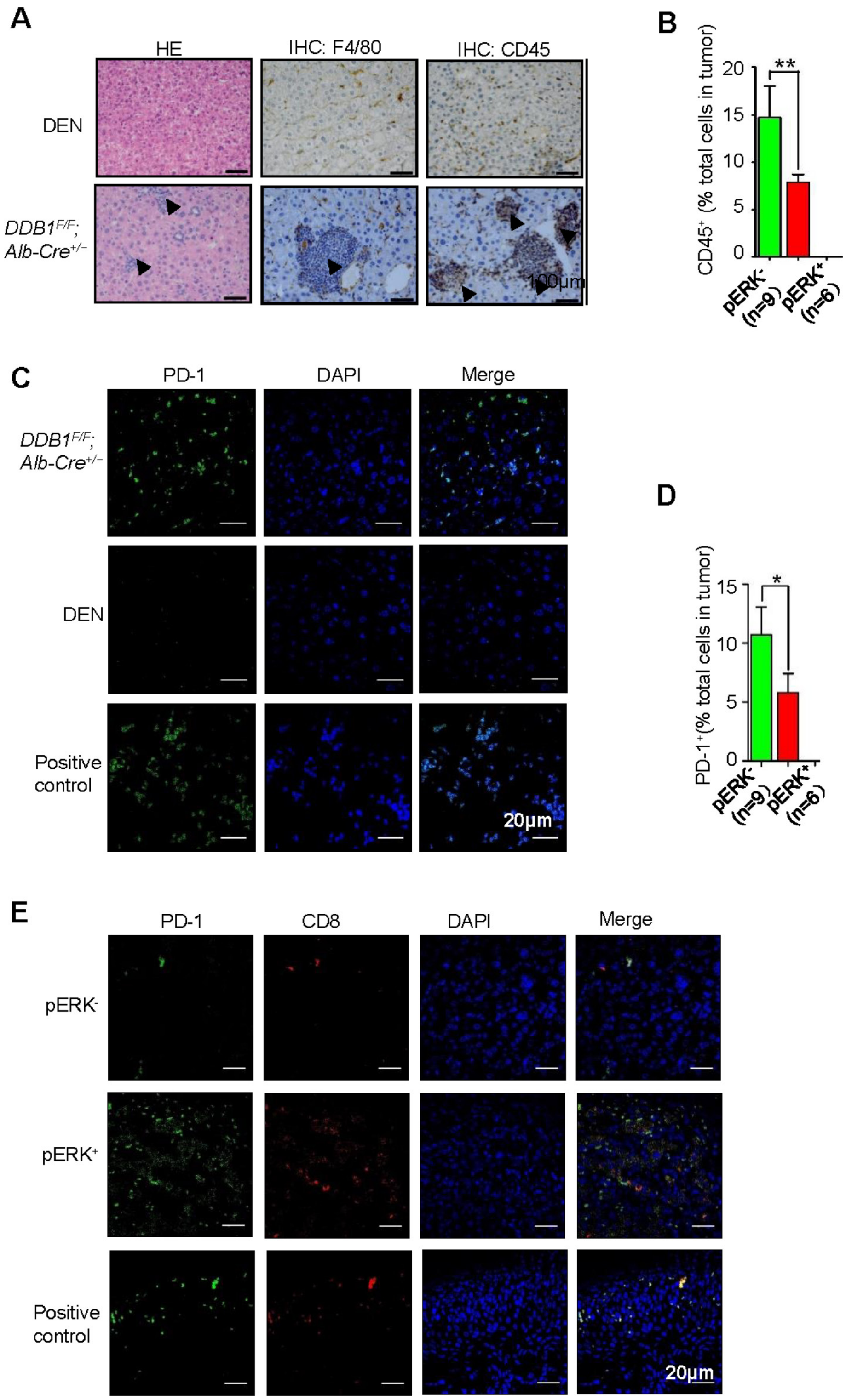

Supplementary Figure S3: Increased inflammation and intratumoral PD-1⁺CD8⁺ T lymphocytes in pERK⁻ HCC samples and mouse liver tumor models. **A.** Representative IHC for CD45 and F4/80 in pERK⁺ and pERK⁻ mouse liver tumor models. Arrowheads indicate inflammatory clusters. **B.** Quantification of CD45⁺ cells in DEN-induced mouse (n=6) and *DDB1*$^{F/F}$*;Alb-Cre*$^{+/-}$ mouse (n=9) liver tumor models. **C.** Representative IF staining for PD-1 in mouse liver tumor models. **D.** Quantification of the percentage of PD-1⁺ cells as in (C) (*P<0.05). **E.** Representative IF staining for PD-1 and CD8 in human HCC samples.

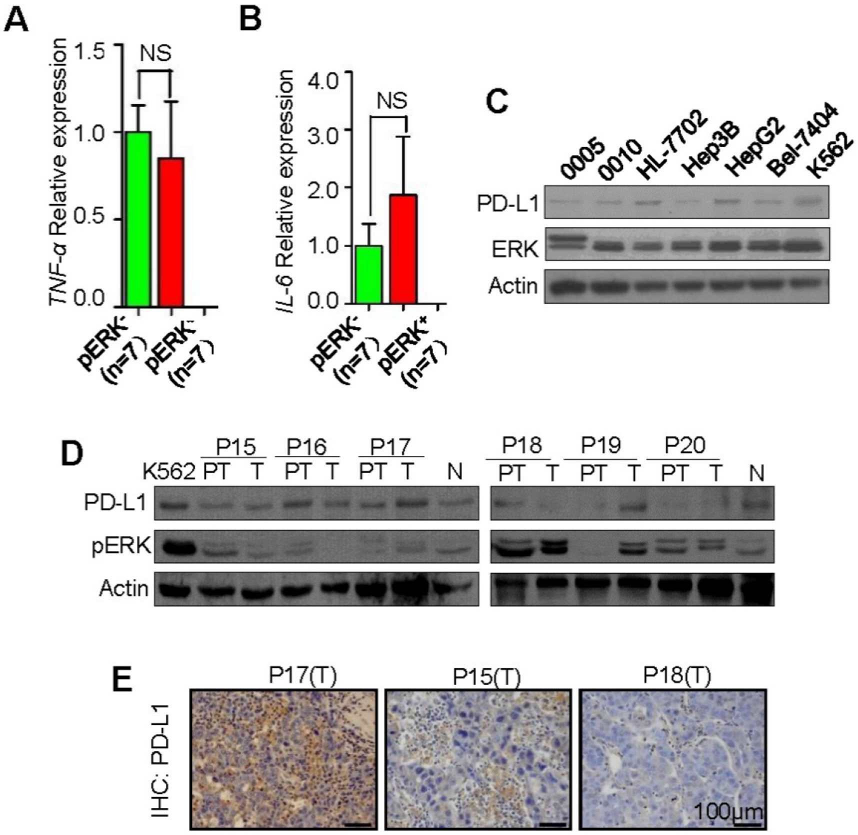

Supplementary Figure S4: Additional characterization of human liver cancer cell lines and HCC samples. Real-time PCR analysis for **A.** *TNF α* and **B.** *IL-6* levels in human tissues. NS, nonsignificant; n=7. **C.** Western blot for PD-L1 in various liver cancer cell lysates. **D.** Western blot for PD-L1 and pERK levels in patient tumor (T) and peritumor (PT) tissues. N, normal liver. **E.** Representative IHC for PD-L1 in HCC sections.

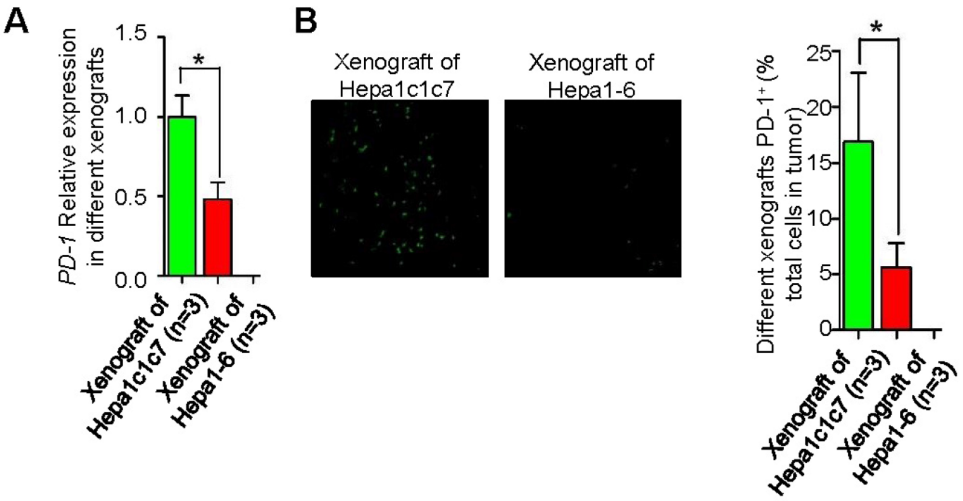

Supplementary Figure S5: Increased intratumoral PD-1^{+} cells in mouse syngeneic liver tumor xenografts. A. Real-time PCR analysis of *PD-1* (*P<0.05; n=3) mRNA levels in syngeneic xenografts developed from two mouse hepatoma cell lines as indicated. **B.** Representative IF staining for PD-1 in these xenografts (left) and quantification of the percentage of PD-1^{+} cells in tumor sections (*P<0.05; n=3) (right).

Supplementary Table S1: Sorafenib inhibition IC50 (μM) of liver cancer cell line proliferation at various days of treatment

Time	HepG2	Bel-7404	Hepa1-6	Hepa1c1c7
Day3	1.044	5.334	4.604	6.534
Day4	0.892	5.378	4.781	3.693
Day5	0.4648	5.161	2.199	5.242
Day6	0.4432	4.522	2.342	3.871

Time	HepG2+U0126	SMMC-7721	QGY-7703	HCC-0010
Day3	1.212	12.4728	7.2174	5.9941
Day4	2.376	3.714	6.6545	2.449
Day5	4.543	6.595	6.0729	2.4685
Day6	/	6.4897	5.8599	1.6741

Supplementary Table S2: Ratio of tumor weight in the sorafenib-treated arm (T) over the vehicle control arm (C) (T/C ratio)

pERK expression	PDX Line	Treatments	Average Tumor Weight (g)	T/C ratio	Group Average
Negative Group	PDX1	Vehicle	3.63	0.79	0.98±0.12
		Sorafenib	2.88		
	PDX3	Vehicle	2.17	0.96	
		Sorafenib	2.09		
	PDX4	Vehicle	2.14	1.19	
		Sorafenib	2.56		
Positive Group	PDX2	Vehicle	1.6	0.23	0.24±0.04
		Sorafenib	0.38		
	PDX5	Vehicle	2.03	0.18	
		Sorafenib	0.37		
	PDX6	Vehicle	1.32	0.32	
		Sorafenib	0.42		

Three $pERK^+$ and three $pERK^-$ PDX models were treated with vehicle control or 30 mg/kg (mpk) (n=5 in each group) and xenograft tumors were removed and weighed at the end of experiments.

Supplementary Table S3: Differential expression of EMT genes in pERK$^+$ and pERK$^-$ xenograft tumors by RNA sequencing

Gene Symbol	Gene Name	Ratio (pERK$^+$/pERK$^-$ tumor)	*P* value
CDH1	*cadherin 1, type 1, E-cadherin (epithelial)*	124:1	6.36E-06
CLDN1	*claudin 1*	7:1	0.02
DSP	*desmoplakin*	15:1	0.0005
KRT8	*keratin 8*	30:1	0.001
TJP2	*tight junction protein 2 (zona occludens 2)*	11:1	0.002
TWIST1	*twist homolog 1 (Drosophila)*	1:15	0.04
CDH12	*cadherin 12, type 2 (N-cadherin 2)*	1:8	0.03
TCF4	*transcription factor 4*	1:10	0.001
BMP7	*bone morphogenetic protein 7*	1:8	0.03

Supplementary Table S4: Patient characterization

Variables	No. of Patients (%)
**No. of patients**	186 (100)
**Age**: Median [range], years	50.05 [17-80]
**Sex**	
Men	157 (84.41)
Women	29 (15.59)
**HBsAg status**	
Negative	33 (17.74)
Positive	153 (82.26)
**HCV status**	
Negative	186 (100)
Positive	0 (0)
**Cirrhosis**	
Absent	73 (39.25)
Present	113 (60.75)
ALT: Median [range], U/L	37.55, 5-256
**Child-Pugh class**	
A	180 (95.40)
B	6 (4.60)
Tumor size: Median [range], cm	4.37 [0.80-13.00]
**Ascites**	
Absent	169 (90.86)
Present	17 (9.14)
**Tumor multiplicity**	
Solitary	175(94.09)
Multiple	11 (5.91)
**Vascular invasion**	
Absent	178 (95.70)
Present	8 (4.30)
**Portal vein invasion**	
Absent	185 (99.46)
Present	1 (0.54)
**BCLC stage**	
0	26 (13.98)
A	157 (84.41)
B	2 (1.08)
C	1 (0.54)
**Micrometastasis**	
Absent	180 (96.77)
Present	6 (3.23)

Abbreviations: ALT, alanine aminotransferase; BCLC, Barcelona Clinic Liver Cancer; HBsAg, hepatitis B surface antigen; HCV, hepatitis C virus.

Supplementary Table S5: Oligonucleotide sequences of primers for quantitative real-time PCR

Target Gene Name	Oligo sequence (5' to 3')	Genomic location
Human-*GAPDH*	CGACCACTTTGTCAAGCTCA	sense
	TTACTCCTTGGAGGCCATGT	antisense
Human-*TNFα*	TGGGGTTTGTGAAACTGTGA	sense
	GTTCCTGCACATTCCCTCTC	antisense
Human-*IL6*	ACTCACCTCTTCAGAACGAATTG	sense
	CCATCTTTGGAAGGTTCAGGTTG	antisense
Human-*PD-1*	ACGAGGGACAATAGGAGCCA	sense
	GGCATACTCCGTCTGCTCAG	antisense
Mouse-*PD-1*	GGGCCTAAGCCTATGTCTCC	sense
	AGGTGTGAAGGAGAGCCAGA	antisense
Human-*PD-L1*	ATTGCAGCTTCACCAGATAGC	sense
	AAAGTTGCATTCCAGGGTCAC	antisense
Human-*CDH1*	ATTTTTCCCTCGACACCCGAT	sense
	TCCCAGGCGTAGACCAAGA	antisense
Human-*TWIST1*	GTCCGCAGTCTTACGAGGAG	sense
	GCTTGAGGGTCTGAATCTTGCT	antisense
Human-*CLDN1*	TCTGGCTATTTTAGTTGCCACAG	sense
	AGAGAGCCTGACCAAATTCGT	antisense
Human-*DSP*	GCAGGATGTACTATTCTCGGC	sense
	CCTGGATGGTGTTCTGGTTCT	antisense
Human-*KRT8*	TGAGGTCAAGGCACAGTACG	sense
	TGATGTTCCGGTTCATCTCA	antisense
Human-*TJP2*	GGGAAGGTCGCTGCTATTGT	sense
	CTCTCGCTGTAGCCACTCC	antisense
Human-*CDH12*	TTTGATGGAGGTCTCCTAACACC	sense
	ACGTTTAACACGTTGGAAATGTG	antisense
Human-*TCF4*	TGCAAAGCCGAATTGAAGATCG	sense
	AGAAGGTCCAATGATTCCATGC	antisense
Human-*BMP7*	GGAACGCTTCGACAATGAGAC	sense
	GCAGGAAGAGATCCGATTCCC	antisense
